# Disentangling EEG Fingerprinting and Sleep Biomarkers Using Generalized Weighted Ordinal Patterns

**DOI:** 10.3390/brainsci16080793

**Published:** 2026-07-28

**Authors:** Cristina Daiana Duarte, Albertina Arlenghi, Francisco Ramiro Iaconis, Gustavo Gasaneo, Claudio Delrieux

**Affiliations:** 1 Departamento de Física, Universidad Nacional del Sur, Bahía Blanca 8000, Argentina; cristina.duarte@uns.edu.ar (C.D.D.); albertina.arlenghi@uns.edu.ar (A.A.); francisco.iaconis@uns.edu.ar (F.R.I.); 2 Instituto de Física del Sur, Consejo Nacional de Investigaciones Científicas y Tecnológicas (CONICET), Bahía Blanca 8000, Argentina; 3 Laboratorio de Ciencias de las Imágenes, Departamento de Ingeniería Eléctrica y Computadoras, Universidad Nacional del Sur-Consejo Nacional de Investigaciones Científicas y Tecnológicas (CONICET), Bahía Blanca 8000, Argentina; cad@uns.edu.ar

**Keywords:** electroencephalography (EEG), sleep stage classification, statistical complexity, fingerprinting, identity confounding

## Abstract

**Background:** Electroencephalographic (EEG) recordings simultaneously contain information about neurophysiological dynamics and subject-specific characteristics. While this duality may enable biomarker discovery and individual identification, it also raises concerns that machine-learning models may achieve high predictive performance by exploiting subject identity rather than physiologically relevant information. **Methods:** In this study, we investigated whether generalized weighted ordinal patterns (GWOP), a statistical-complexity representation incorporating both temporal ordering and amplitude fluctuations, support sleep-stage classification while minimizing identity-related confounding. Sleep EEG recordings from 31 healthy subjects were segmented into 30-s epochs and represented using 3150 GWOP features derived from multiple embedding dimensions, time delays, and entropic indices. XGBoost classifiers were evaluated under intra-subject and inter-subject validation schemes to quantify the impact of EEG fingerprinting on sleep-stage classification performance. An additional subject-identification analysis was conducted using the same feature representation. **Results:** Sleep-stage classification generalized well to previously unseen subjects, with accuracy decreasing only from 79.2% to 75.8% between intra-subject and inter-subject evaluations. Feature-importance analysis using SHAP revealed an almost perfect correspondence between the features driving classification in both validation schemes (Spearman ρ=0.998). **Conclusions:** While this suggests that the models effectively generalize across subjects without being heavily confounded by individual identities, it indicates a framework of partial separation rather than complete orthogonality across the global feature space. In contrast, GWOP features also supported subject identification with 63.9% accuracy across the 31 individuals, demonstrating that GWOP preserve substantial fingerprinting information. The most informative features for subject identification showed little overlap with those governing sleep-stage classification, suggesting a partial separation between identity-related and biomarker-related information within the same feature space. These findings suggest that EEG fingerprinting and biomarker extraction are not necessarily competing objectives and support GWOP-based statistical-complexity measures as a promising proof-of-concept framework for robust sleep EEG analysis, serving as a foundation for future scale-up precision-neuroscience applications.

## 1. Introduction

Predictive analysis applies multivariate statistical models and machine learning (ML) to leverage information from feature sets with the purpose of forecasting unknown, prognostically valuable outcomes. This methodological approach gained prominence in biomedical research, where a recent influx of research results is showing its potential to yield biomarkers capable of impacting both clinical practice and public health. Electroencephalography (EEG)—a widely utilized technique for characterizing neural activity—constitutes a particular focal point of extensive investigation aimed at identifying specific biomarkers associated with neurological and psychiatric disorders. A salient challenge in these investigations is an adequate identification and quantification of underlying confounding biases that may arise [1]. This is especially the case in EEG signals, where highly individualized patterns of brain activity are sufficiently stable to enable subject identification across recording sessions, a phenomenon known as EEG fingerprinting [2]. These subject-specific signatures may arise from a combination of anatomical, physiological, and functional factors, including stable oscillatory profiles, connectivity patterns, and large-scale network dynamics [3]. The existence of such information offers valuable opportunities for personalized medicine and longitudinal monitoring. However, it also introduces methodological challenges in finding adequate biomarkers in EEG research, specifically if these are sought after using machine learning techniques. In particular, strong individual components present in EEG recordings can give rise to *identity confounding*, a form of data leakage in which predictive models inadvertently learn subject-specific rather than disease-related features [4]. This issue is especially relevant in studies where inadequate train-test partitioning may allow information pertaining to the same individual to appear in both datasets. Under these conditions, models can achieve inflated classification performance in using stable personal characteristics that may be unrelated to the actual pathology under investigation.

Conceptually, fingerprinting and identity confounding represent two manifestations of the same underlying property, that is, the presence of robust subject-related information in the EEG signal [5]. As such, this may be either advantageous or problematic depending on the specific task at hand. Whereas fingerprinting explicitly seeks to quantify and leverage this information, identity confounding becomes problematic when the goal is to detect pathological, physiological, or behavioral biomarkers that generalize across individuals [6]. Consequently, rigorous subject-wise validation procedures have become essential to warrant that reported classification performance reflects genuinely generalized disease-specific features rather than subject-based recognition. This consideration is particularly critical in the study of sleep disorders, where individual variability present in overnight EEG activity recording sessions can be substantial and may exceed the magnitude of pathology-related effects [7,8,9]. In these situations, the use of parts of the same session in the train-test split potentially compromises the generalizability and reproducibility of the results [4,10].

From the brain sciences standpoint, however, there is an intriguing possibility that some biomarkers are expressed through the fingerprint itself [11]. For example, a neurodegenerative disorder may not simply add an abnormal EEG pattern, but instead it may progressively alter the individual’s characteristic dynamical signature. In such cases, the most sensitive biomarker may be the deviation of a person’s current EEG from their own historical fingerprint rather than from a population average. This idea is particularly worth pursuing in sleep EEG research, because sleep recordings are rich, repeatedly acquired, and strongly individualized [6]. Consequently, EEG-based sleep research may increasingly focus on distinguishing stable personal traits, transient physiological states, and pathology-related changes, rather than treating fingerprinting solely as a nuisance circumstance to be eliminated, as is mostly the case with current feature-based learning models. In other words, new signal analysis techniques are required with the capacity of extracting latent information in which both population-wise trends and individuals’ characteristics are orthogonally represented in the model’s feature space.

To address this challenge, here we focus on the potential applicability of statistical complexity, in particular on the use of ordinal patterns, as adequate features in sleep EEG signals [12,13,14]. EEG signals are known to contain stable individual characteristics that arise from anatomical, physiological, and functional properties of the brain, enabling reliable identification of subjects across recording sessions. At the same time, sleep EEG recordings also capture state- and disease-related alterations related to insomnia, obstructive sleep apnea, narcolepsy, or other sleep pathologies. Consequently, a single EEG signal may simultaneously encode information about individual identity and pathological status. We evaluated whether ordinal-pattern features derived from Generalized Weighted Permutation Entropy (GWPE) [15] provide robust representations for EEG sleep-stage classification without producing strong subject-specific fingerprinting effects. To investigate this, we compare sleep stage classifier performance under two validation schemes: an intra-subject (“record-wise”) setup, where epochs from the same subjects appear in both training and testing sets, and a subject-independent (“subject-wise”) setup, where unseen subjects are used exclusively for testing. In a second analysis, we trained a subject classifier using the same features extracted across all sleep stages. We found that the presence of subject-specific EEG complexity signatures does not preclude the identification of clinically relevant biomarkers, in this case, supervised labeled sleep stages. Our results show that both sources of latent information may coexist within the same recordings. These results were assessed using SHapley Additive exPlanations (SHAP) to evaluate the partial dependency of the models with respect to feature contributions [16]. This perspective suggests that fingerprinting and biomarker discovery should not necessarily be viewed as competing objectives, but rather as complementary aspects of brain dynamics operating at different layers of organization.

## 2. Materials and Methods

### 2.1. Datasets

We analyzed the polysomnographic records available in the Sleep-EDF Database Expanded [17], a publicly accessible resource distributed via PhysioNet [18]. This repository comprises whole-night polysomnographic recordings derived from two independent protocols: the Sleep Cassette Study and the Sleep Telemetry Study. The Sleep Cassette Study was designed to evaluate age-related alterations in sleep architecture among healthy, unmedicated participants. Each subject contributed two consecutive overnight recordings, yielding a total of 153 polysomnographic sessions. The Sleep Telemetry Study investigated the effects of temazepam on sleep physiology in healthy individuals reporting mild sleep-onset difficulties. This protocol includes 44 recordings obtained from 22 subjects. Both studies encompass electroencephalography (EEG), electrooculography (EOG), and chin electromyography (EMG) signals. EEG data were acquired using the Fpz-Cz and Pz-Oz derivations, with a sampling rate of 100 Hz, and partitioned in nonoverlapping segments of 30 s (called “epochs”).

Expert sleep-stage annotations were provided for each epoch. In the present work, we exclusively utilized the Fpz-Cz EEG channel. For the Sleep Cassette Study, we retained only the first night of recording per subject. For the Sleep Telemetry Study, we included only recordings from subjects who did not receive active medication (i.e., placebo or no-treatment conditions). The final analytical sample comprised male and female participants aged between 18 and 40 years (mean age: 27.59, standard deviation: 4.74). A total of N=31 subjects were included in the analysis, comprising 18 females (mean age: 28.6, standard deviation: 5.3) and 13 males (mean age: 26.0, standard deviation: 4.0). Epochs containing recording artifacts were removed from the analysis. Specifically, epochs in which the EEG signal remained constant over time were excluded, since this behavior indicates a failure in signal acquisition rather than physiological brain activity. A temporal windowing function was applied to retain only epochs within a biologically relevant sleep window. Specifically, the analysis was restricted to the 30 min before the first non-Wake epoch and the 30 min after the last non-Wake epoch of each recording. This removes artifactual wakefulness at the edges of the recordings and focuses the analysis on the actual sleep period. No digital filtering or downsampling was applied to the raw EEG recordings to preserve the original signal dynamics, since linear preprocessing reduces noise at the expense of filtering tiny fluctuations that may be significant in nonlinear signal analysis. The proposed framework presented below performs a multi-scale analysis through two intrinsic parameters that evaluate the relative contribution of signal fluctuations at different amplitudes and temporal scales. Consequently, the proposed features enable an EEG analysis directly from the original recordings, without requiring specific acquisition-dependent parameters. This also supports the robustness and generalizability of the results.

### 2.2. Ordinal Pattern Analysis

The characterization of these EEG signals was conducted using the analysis of Ordinal Patterns (OPs), a robust non-linear methodology originally introduced by Bandt and Pompe [12] to evaluate permutation entropy patterns in signals and time series. This framework is grounded in the symbolic representation of time series through the ranking of amplitude values within successive (and likely delay-embedded) vectors, thereby enabling the quantification of local temporal orderings in the signal. A widely acknowledged advantage of this approach lies in its capacity to effectively capture underlying dynamic structures and phase-space trajectories in chaotic dynamical systems, distinguishing apart stationary regimes from transient deterministic behavior (chaotic) and randomness [19]. Consequently, OPs preserve the intrinsic temporal correlations of time series while remaining computationally efficient and resilient to monotonic transformations of the data. This has been advantageous in the analysis of neurophysiological processes without imposing strict stationarity assumptions on the signal. In the context of sleep EEG analysis, this framework facilitates the characterization of nonlinear, nonstationary dynamical properties, such as complexity, regularity, and information content, across distinct sleep stages and physiological states [20].

#### 2.2.1. Ordinal Pattern Construction

For a given EEG time series ${\left\{x_{t}\right\}}_{t=1}^{T}$, symbolic mapping is performed based on the relative order of data points within vectors of dimension *D* (representing the embedding dimension or window length). These vectors are constructed by introducing a time delay or lag (τ) between consecutive samples of the pattern, defined as follows:

(1) $$X_{s}^{\left(D,\tau \right)}=\left(x_{s},x_{s+\tau },\ldots ,x_{s+(D-1)\tau }\right).$$

The vector $X_{s}^{\left(D,\tau \right)}$ is represented with an OP π, which is determined by the permutation that sorts the amplitude values of the segment in ascending order. In particular, let $\left(r_{0},r_{1},\ldots ,r_{D-1}\right)$ be the unique permutation of (0,1,…,D−1) such that:

$$x_{t+r_{0}\tau }\leq x_{t+r_{1}\tau }\leq \cdots \leq x_{t+r_{D-1}\tau }.$$

In the event of ties (i.e., identical amplitude values within the same embedding vector due to signal quantization), ranks are resolved deterministically using chronological ordering, assigning the lower rank to the temporally preceding sample. This corresponds to the stable sorting convention (lexicographic resolution). Then the associated ordinal pattern is defined as $\pi =(r_{0},r_{1},\ldots ,r_{D-1})$. Each sample segment of length *D* maps injectively onto one of the D! possible OPs, providing a symbolic encoding of its local temporal structure. We analyzed window lengths of D=3,4,and5 points and lag values of τ=1,2,and3. Each window length yields a total of D! possible permutations (e.g., 6, 24, and 120 OPs, respectively), which define the symbolic alphabet of the underlying brain dynamics. Hence, selecting a larger *D* enables wider variety of local amplitude rank orderings within the delay-embedded time series. This increased pattern diversity facilitates a more detailed representation of the signal’s nonlinear dynamics, enhancing sensitivity to subtle temporal variabilities, structural complexities, and regime-specific neural signatures.

In a given signal, each segment will be injected into a unique OP embedding, and thus each of the $\pi_{i}$ OPs (i∈D!) will be represented with a relative frequency $p(\pi_{i})$ (i.e., the empirical probability of observing this OP within the delay-embedded time series). The empirical probability distribution over the D! possible patterns constitutes a feature set that enables advanced signal analysis and characterization. In the context of sleep EEG analysis, a sufficiently large *D* supports a richer and more detailed characterization of neurophysiological states. Varying τ, in turn, functions as a downsampling filter, capturing rapid biological rhythms when τ=1, or isolating lower-frequency sleep dynamics (such as delta and theta waves) as the delay increases. It is worth noting that both parameters (*D* and τ) operate independently, and that the set of OPs produced under different combinations are not redundant (i.e., for a given signal and a given τ, the six OPs produced with D=3 are likely to be statistically independent to the 120 OPs produced with D=5). In other words, the combined use of different signal embeddings may enrich the feature space significantly, allowing the development of more advanced ML-based analyses.

#### 2.2.2. Generalized Weighted Ordinal Patterns (GWOP) and Entropic Index *q*

So far, the signal embedding technique described above relies exclusively on sequential ordering, and is thus invariant under monotonic transformations, and neglects amplitude fluctuations. Consequently, either low-intensity background noise or high amplitude synchronized waves—such as K-complexes or delta wave bursts—may be handled identically. To overcome this limitation, we adopted a generalization of the Bandt-Pompe schema, namely the Generalized Weighted Permutation Entropy (GWPE) formalism [15,21]. Under this framework, patterns within the signal do not contribute uniformly to the analysis. Instead, each occurrence is weighted by its local variance raised to an exponent *q*, usually known as *entropic index*. The generalized probability of occurrence $p(\pi_{i},q)$ for each OP $\pi_{i}$ is then calculated as follows:

(2) $$p\left(\pi_{i},q\right)=\frac{\sum_{s\in S,\pi_{s}=\pi_{i}}w_{s}^{q/2}}{\sum_{s\in S}w_{s}^{q/2}}.$$

Here, $w_{s}$ denotes the variance (amplitude fluctuation) of the analyzed segment. Positive *q* values emphasize the contribution of segments with large amplitude fluctuations, which is crucial for isolating high-energy macro-structures such as deep-sleep delta waves (N3 stage). Negative *q* values, in turn, favor small amplitude fluctuations, enabling the study of baseline noise or subtle transitions during micro-arousals and REM sleep. When q=0, the model consistently reduces to the standard unweighted Bandt-Pompe ordinal probability distribution.

As already mentioned, EEG recordings were segmented into 30-s epochs. Each epoch was labeled according to its corresponding sleep stage. For each epoch, the GWOP were computed using embedding dimensions D=3,4, and 5 using time delays τ = 1, 2, and 3 and entropic indices *q* from −1 to 5. These values were selected based on the robustness and significance reported in previous studies [20,22]. These features will be used to develop ML-based analyses to evaluate the extent to which GWOP provide reliable representations for the classification of EEG-derived sleep stages while avoiding strong subject-specific fingerprinting. The total amount of features is summarized in Table 1.

### 2.3. Experimental Design

**Figure 1:**
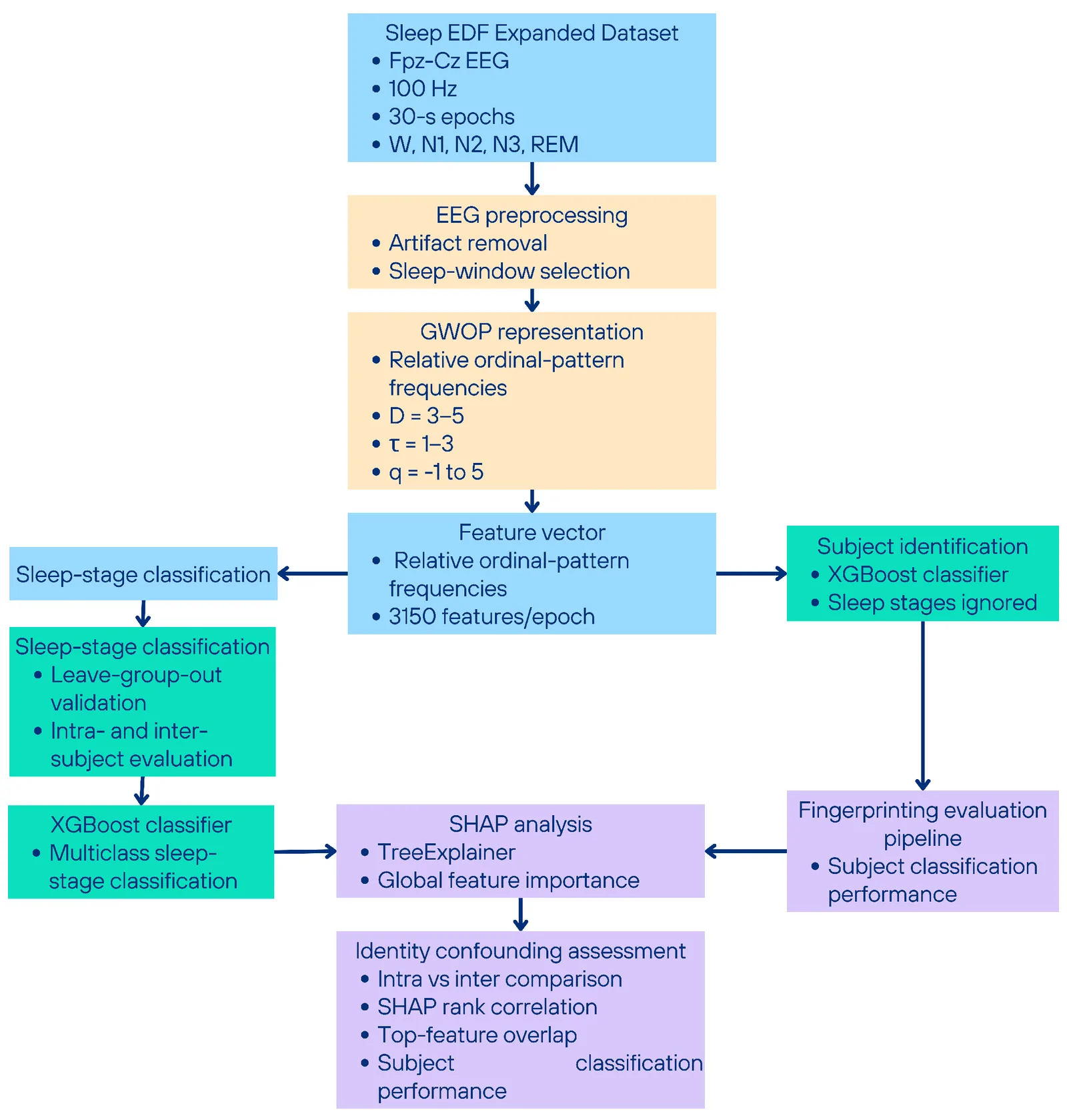
Processing workflow performed in this study.

To evaluate the influence of subject-specific fingerprinting on the proposed classification model, two complementary evaluation schemes were implemented. First, sleep-stage classification performance was compared under intra-subject and inter-subject conditions, i.e., when the classifier was tested on epochs from subjects included in the training set versus epochs from previously unseen subjects. This analysis was designed to evaluate whether subject-specific characteristics contribute to sleep-stage classification performance beyond sleep-related dynamics. Second, a subject-identification analysis was performed using the same feature representation, with subject identity as the classification target instead of the sleep stage. This experiment provided a direct evaluation of the extent to which the extracted features encode subject-specific information. The entire workflow is illustrated in Figure 1.

#### 2.3.1. Classification Model

As already mentioned, sleep stage classification models were trained and assessed under two distinct scenarios: an intra-subject condition, where the test set comprised data from subjects present during training (internal split), and an inter-subject condition, where the test set consisted of data from four previously unseen randomly selected subjects. A leave-group-out validation procedure was conducted across 24 trials to evaluate performance under these two conditions. To ensure reproducibility, the random selection of held-out groups was performed using a fixed random seed (42). Each of the 24 selected groups was unique, with no two trials sharing the same combination of four held-out subjects. Across the 24 trials, the number of times an individual subject was assigned to a held-out group ranged from 0 to 7 (mean = 3.10±1.60), with three subjects appearing in none of the held-out groups. In each trial the experimental procedure was performed as follows:

1. Features were extracted from all 30-s EEG epochs corresponding to the 31 subjects included in the study (see Section 2.2.2).
2. For each trial, four subjects were randomly selected and excluded from the training process. These subjects formed the inter-subject test group.
3. The remaining 27 subjects were used for model development. Their epochs were randomly divided into training and test subsets using a 85%/15% split. This split was used for intra-subject evaluation, since epochs from the same subjects were present in both subsets.
4. The same trained model was then evaluated on all epochs belonging to the four held-out subjects. This evaluation corresponds to the inter-subject validation scheme, since the model was tested on subjects not seen during training.

#### 2.3.2. Subject Identification

To further assess the presence of subject-specific information within the GWOP representation, an additional subject-identification analysis was conducted regardless of sleep stages using the same GWOP feature set. In contrast to the sleep-stage classification task, the objective of this analysis was to determine whether these features contain sufficient information to discriminate individual subjects.

To contextualize the identity-related information captured by the GWOP representation, the subject-identification analysis was repeated using a baseline feature set composed of ten conventional spectral descriptors. For each 30-s epoch from the same Fpz-Cz channel, the baseline included the absolute and relative powers in the delta (0.5–4 Hz), theta (4–8 Hz), alpha (8–13 Hz), sigma (12–15 Hz) and beta (15–30 Hz) frequency bands. Both representations were evaluated using identical preprocessing, class-balancing, data-partitioning and hyperparameter-optimization procedures, ensuring that any performance difference could be attributed solely to the feature representation.

### 2.4. Classification Algorithm

Sleep stage classification and subject identification models were developed using the XGBoost algorithm based on the 3150 previously described GWOP features. Class balancing and hyperparameter tuning were performed using RandomizedSearchCV from the scikit-learn library [23]. For sleep stage classification, models were trained on epochs from the 27 subjects included in the training set of each of the 24 cross-validation trials. To mitigate class imbalance, sleep stage classes were balanced on a per-subject basis. Specifically, for each subject, the number of epochs in each sleep stage was randomly downsampled to match the size of the minority class. As a result, the total number of epochs available for training varied across trials, depending on the specific composition of subjects in each training set. Following class balancing, the data for each trial were split into training (85%) and testing (15%) sets. The mean number of training epochs was $N_{\mathrm{train}}=6185.4\pm 238.5$ (mean ± standard deviation). The mean number of epochs used for intra-subject testing was $N_{\mathrm{test},\mathrm{intra}}=1091.9\pm 42.1$, whereas the mean number of epochs used for inter-subject testing was $N_{\mathrm{test},\mathrm{inter}}=4296.9\pm 309.8$. The larger number of epochs in the inter-subject testing set is due to the inclusion of all available epochs from the four held-out subjects, whose data were not used during training.

For hyperparameter tuning, an initial search was first conducted using a random intra-subject split. This preliminary optimization used 10-fold cross-validation and 75 random parameter combinations. The resulting hyperparameters were then used as the starting point for the optimization performed during the 24 experimental trials. For each trial, a second hyperparameter optimization was carried out using 7-fold cross-validation and 20 random parameter combinations. Model performance was evaluated using classification accuracy.

Feature contributions to the XGBoost sleep stage classification models were evaluated using SHapley Additive exPlanations (SHAP). SHAP values were computed with the TreeExplainer algorithm [24] for each trained model on a random subset of 1000 epochs. As the classifier addresses a multiclass problem, aggregate feature importance was defined as the mean absolute SHAP value, averaged across all sleep stages and the 24 experimental trials. Subsequently, stage-specific feature importance patterns were examined independently for each sleep stage. To compare feature relevance across known and unseen subjects, separate analyses were conducted for intra-subject and inter-subject evaluation conditions. Finally, feature rankings were assessed using Spearman correlation and top-feature overlap to quantify the stability, consistency, and potential subject specificity of the GWOP representations.

For the intra-subject evaluation, class balancing was performed prior to the train-test split. This test set, despite representing an artificial distribution, forces the classifier to optimize for subtle morphological transitions (e.g., N1 vs. REM) rather than exploiting majority class prevalence. Consequently, this guarantees that the subsequent feature importance analysis (via SHAP values) captures genuine neurophysiological fingerprints instead of artifactual class base-rates. Conversely, the inter-subject validation naturally retains the original, unbalanced physiological distribution to assess real-world generalizability.

For the subject identification analysis, the classifier was trained using GWOP features extracted from epochs belonging to all sleep stages and all 31 subjects. To mitigate class imbalance, the number of epochs for each subject was randomly reduced to match the size of the minority class. After balancing, a total of 24,552 epochs were available for analysis, corresponding to 792 epochs per subject. The dataset was randomly partitioned into training and testing subsets using an 85%/15% split, resulting in 20,869 training epochs and 3683 testing epochs. Hyperparameter optimization was performed using RandomizedSearchCV with 7-fold cross-validation and 100 randomly sampled hyperparameter combinations. The optimal hyperparameters were subsequently used to train the final subject-identification model.

## 3. Results

In this section we present the main results obtained from the intra- and inter-subject sleep stage classifiers, as well as the findings from the subject identification analysis. A substantial part of this presentation is devoted to investigating the extent to which the GWOP features may exhibit a detrimental fingerprinting effect. Accordingly, special attention was paid to identifying which of the 3150 features were the most important within each model according to SHAP analysis, and the extent to which these features overlap.

### 3.1. Sleep Stage Classification

**Figure 2:**
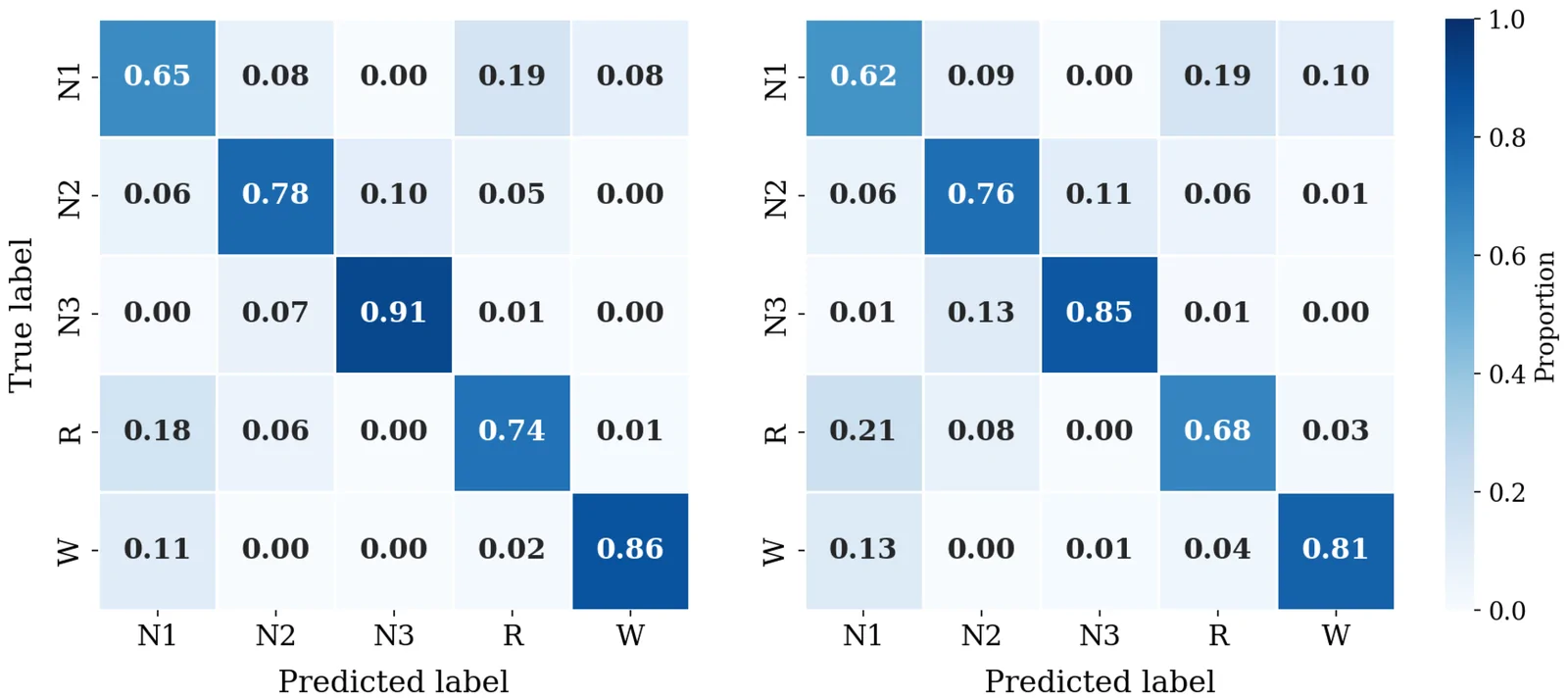
Normalized confusion matrices averaged over 24 trials for intra-subject validation (**left**) and inter-subject validation (**right**).

**Figure 3:**
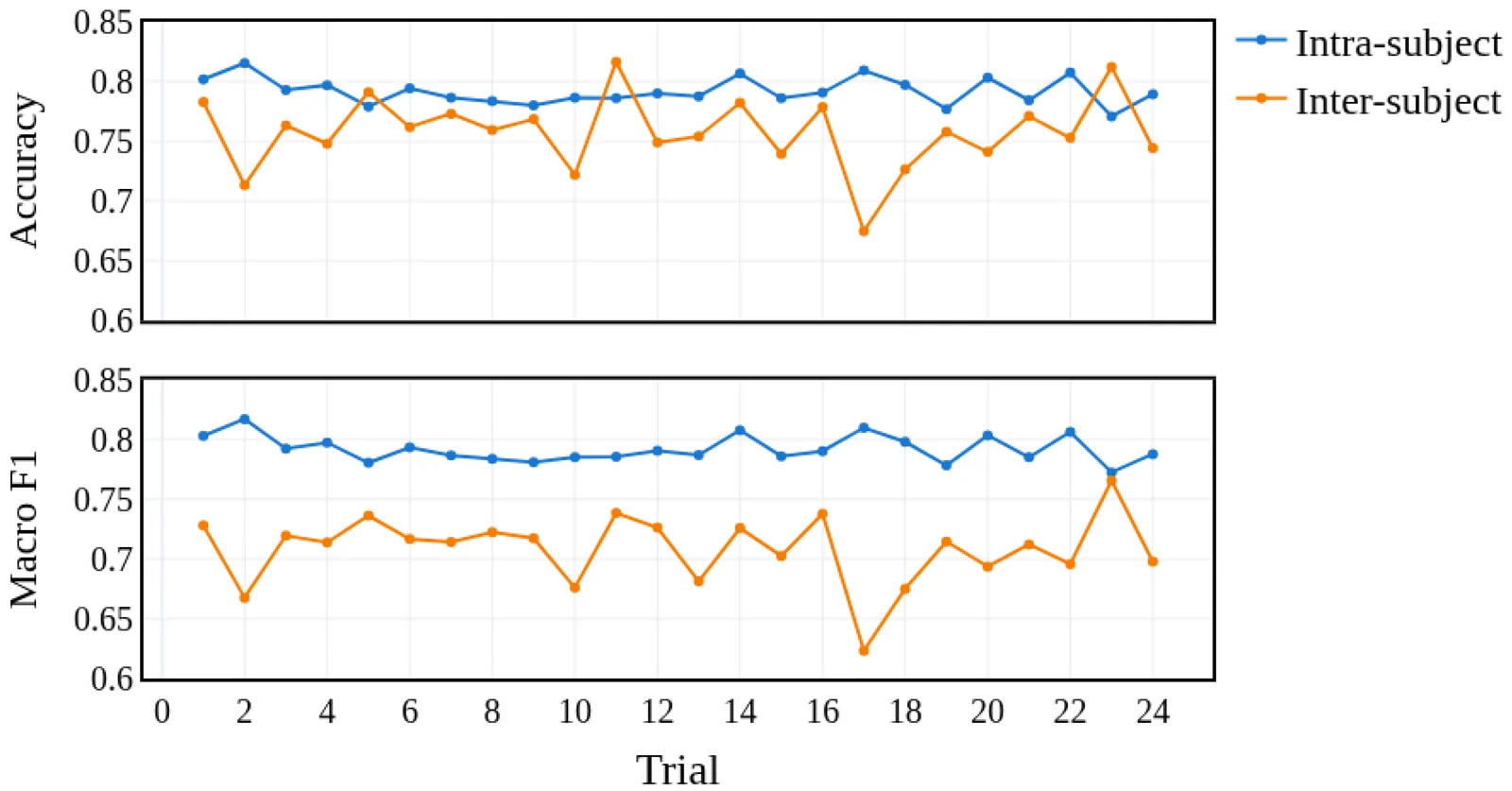
Accuracy and Macro-F1 scores obtained across the 24 validation trials for the intra-subject and inter-subject evaluation schemes.

Table 2 provides a comprehensive summary of the mean accuracy, balanced accuracy, macro F1-score, and Cohen’s κ for sleep stage classification performance under both intra-subject and inter-subject conditions, averaged across 24 independent trials. In addition, Wilcoxon signed-rank tests were performed to compare the two conditions, and the corresponding *p*-values are also reported in Table 2. Table 3 reports the mean precision, recall and F1-score for each sleep stage under both validation conditions. The mean value and standard deviation of the optimal hyperparameters distribution across the 24 trials are summarized in Table 4. Figure 2 displays the normalized average confusion matrices for the two conditions, highlighting the fact that systematic differences in classification patterns are not present. Wilcoxon signed-rank tests indicated statistically significant differences between the intra-subject and inter-subject conditions across all evaluated metrics (all p<0.001). Bootstrap-derived 95% confidence intervals for the performance differences are also reported in Table 2. All evaluated global metrics slightly declined when moving from the intra-subject to the inter-subject setting. The most pronounced drop was observed for the macro-averaged F1 score (Δ = 0.084), followed by Cohen’s κ (Δ = 0.061). Smaller decreases were noted for balanced accuracy and mean accuracy, which highlights the relative low sensitivity of the F1 score to cross-subject variability. Overall, the intra-subject condition yields higher performance across most sleep stages, with the exception of N2, where inter-subject precision exceeds its intra-subject counterpart. The largest performance gap between conditions is observed in N1, where inter-subject precision drops by 0.35 relative to the intra-subject condition. This reduction in precision is reflected in the F1-score, which falls from 0.646±0.027 in the intra-subject condition to 0.390±0.050 in the inter-subject condition. The per-trial evolution of accuracy and macro F1 score is illustrated in Figure 3, which reveals only minor trial-to-trial fluctuations and confirms the robustness of the observed performance across individual runs.

To investigate whether the classifier relied on subject-specific identity signals, the top-*N* intra and inter SHAP feature sets were compared for increasing values of *N* (Table 5). Features that are present only in the intra ranking are potential candidates for fingerprinting, as their presence would suggest that the model uses subject identity rather than sleep stage information to make predictions about subjects that have been seen before. The stability of SHAP feature rankings between trials was assessed by computing the Spearman rank correlation coefficient for all 242=276 trial pairs. For each pair of trials, the correlation (ρ) measures how consistently the 3150 features are ranked by importance: ρ=1 indicates identical rankings, while ρ=0 indicates no agreement. The results are summarized in Table 6.

**Figure 4:**
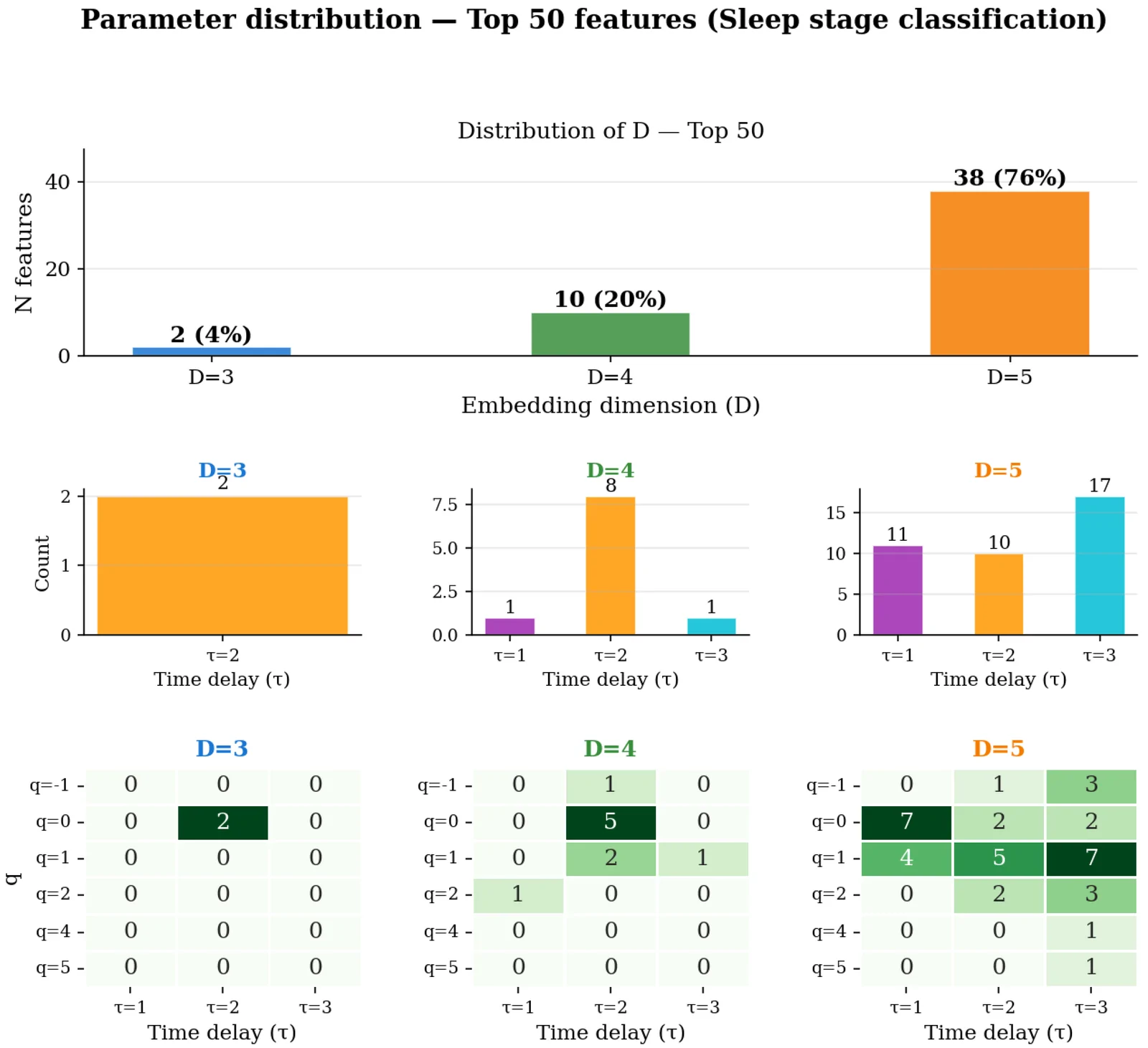
Distribution of embedding dimension (*D*), time delay (τ), and entropic index (*q*) among the top-50 GWOP features ranked by global mean absolute SHAP value for sleep stage classification. The (**upper**) panel shows the number of features for each value of *D*; the (**middle**) panels show the distribution of τ within each group of *D*; and the (**lower**) panels show a heatmap of the joint distribution of *q* and τ for each value of *D*. Methodologically, the strong predominance of higher embedding dimensions (D=5, 76%) and extended time delays (τ=3) indicates that generalizable sleep-stage classification relies heavily on capturing lower-frequency macrostructural neural rhythms, such as delta and theta waves. The wide distribution across multiple *q* values reflects the model’s need to balance both high-amplitude synchronized waveforms and subtle low-amplitude transitions to accurately differentiate global physiological states.

Table 7 lists the ten highest-ranked features by global mean absolute SHAP value. Notably, the ranking is identical under both test conditions, and the importance values coincide to four decimal places. The Spearman rank correlation between the full intra- and inter-subject importance vectors (across all 3150 features) was ρ=0.998 (p<0.001), indicating near-perfect agreement. Figure 4 shows that the distribution of *D* and τ among the top-50 features for intra- and inter-subject conditions.

### 3.2. Subject Classification Analysis

**Figure 5:**
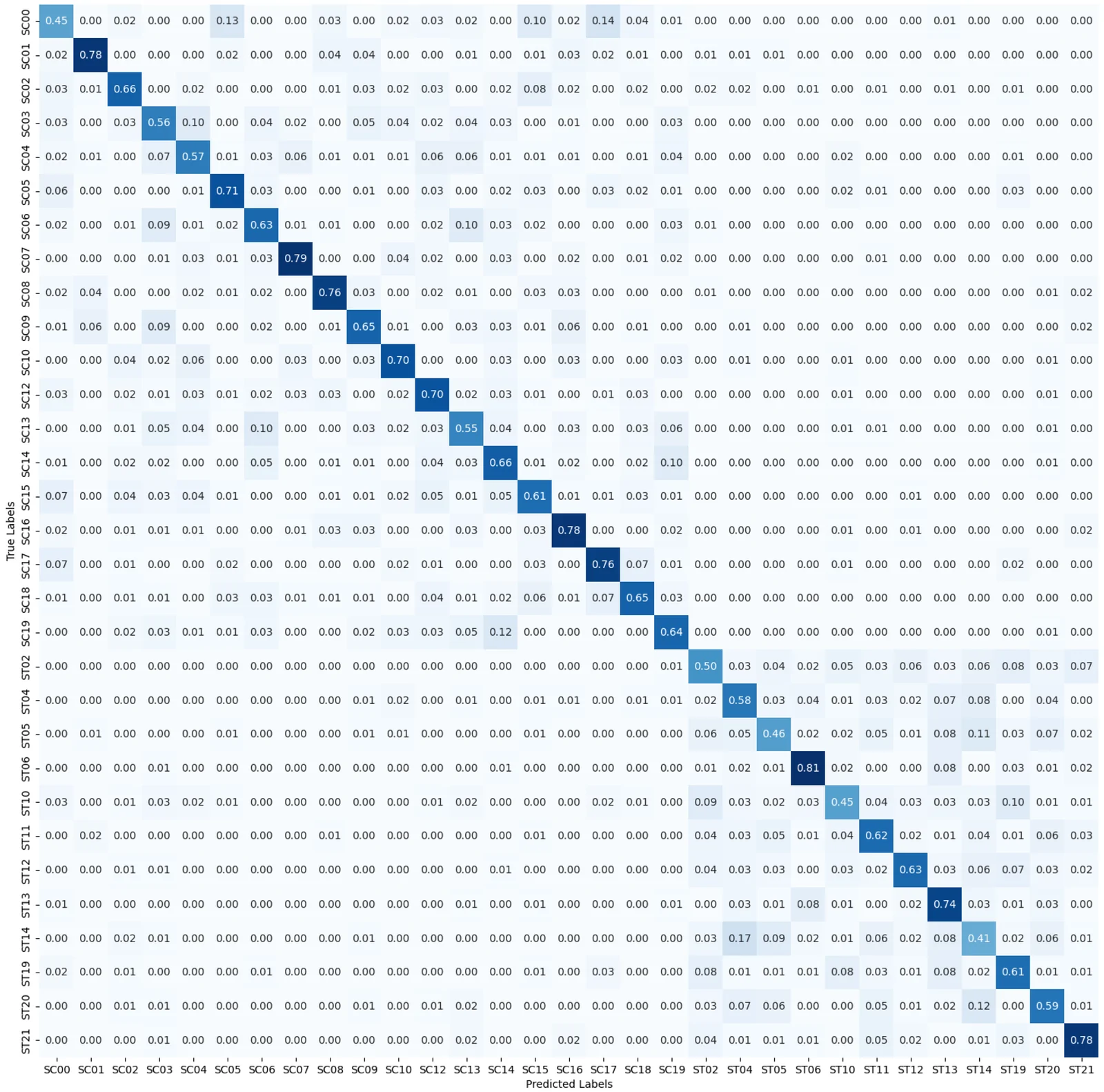
Confusion matrix for subject classification.

To directly evaluate whether subject-specific information is present in the GWOP feature space, we conducted a subject-identification analysis using the same feature representations that were employed for sleep-stage classification. This analysis was performed across all available epochs, regardless of their assigned sleep stage, to ensure a comprehensive assessment of subject discriminability. The resulting performance is visualized in Figure 5, which presents the normalized confusion matrix for subject identification. Table 8 shows the evaluation metrics for the subject-identification classifier for the GWOP feature representation and the baseline representation based on spectral features. The results suggest that GWOP encodes richer subject-specific characteristics than conventional spectral features, resulting in an increase in identification accuracy from 0.432 to 0.639. During hyperparameter optimization, the best-performing model achieved a Macro-F1 standard deviation of 0.006 across the seven cross-validation folds. Table 9 summarizes the corresponding hyperparameter values.

**Figure 6:**
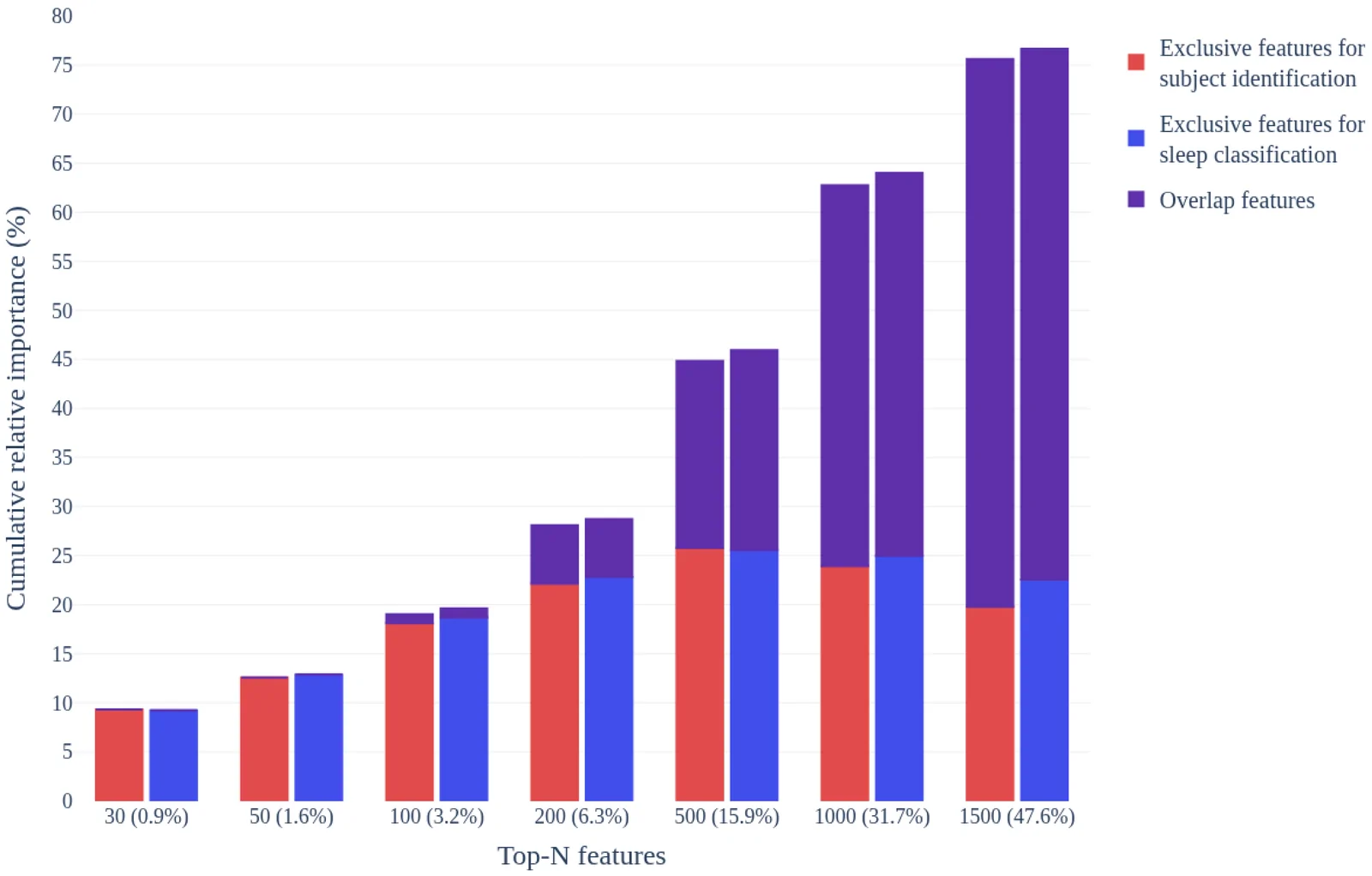
Cumulative feature importance for sleep stage classification and subject identification for the N-top features.

**Figure 7:**
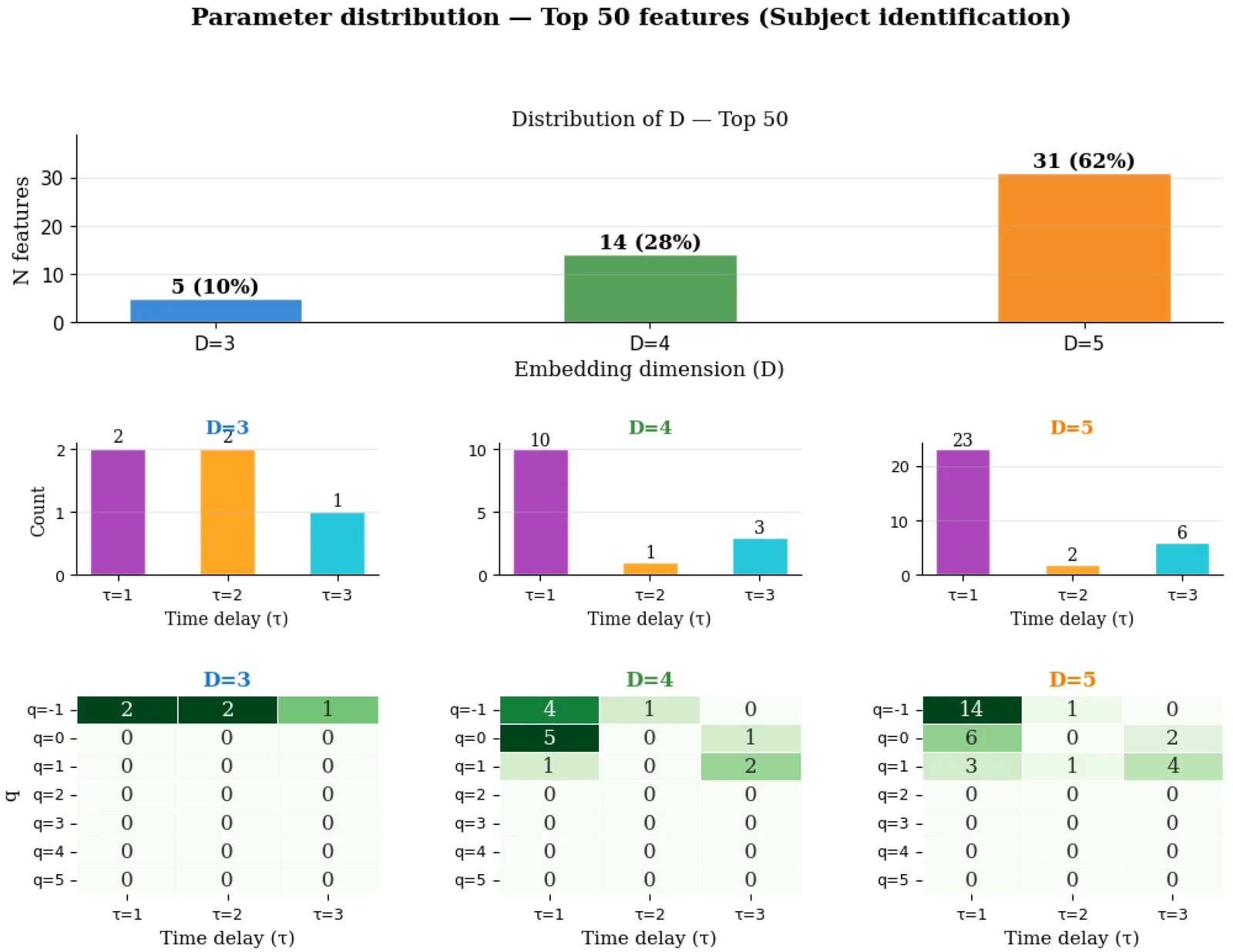
Distribution of embedding dimension (*D*), time delay (τ), and entropic index (*q*) among the top-50 GWOP features ranked by global mean absolute SHAP value for subject classification. The (**upper**) panel shows the number of features for each value of *D*; the (**middle**) panels show the distribution of τ within each group of *D*; and the (**lower**) panels show a heatmap of the joint distribution of *q* and τ for each value of *D*. In contrast to sleep scoring, the sharp concentration of top identity features at minimal time delays (τ=1) combined with negative or zero entropic indices (q=−1, q=0) provides strong methodological evidence that individual EEG fingerprints are primarily encoded within fast, high-frequency microstructural dynamics and baseline noise distributions. This structural pattern underscores why the identity signature remains remarkably stable across the night without confounding the macrostructural signatures required for sleep-stage tracking.

To further investigate whether the GWOP features encode subject-specific information independently of sleep stage, we compared the SHAP-based feature rankings derived from the sleep-stage classifier with those obtained from the subject-identification classifier. This comparison allowed us to assess the degree of overlap between the most significant GWOP features for each task. Table 10 reports the overlap between the top-*N* most important features for sleep-stage classification and those for subject identification. Figure 6 illustrates the cumulative relative feature importance for both classifiers. Features exclusive to subject identification, exclusive to sleep-stage classification, and shared between both models are distinguished by color. For N<30, the overlap between the two feature sets is zero, implying that most important features for sleep-stage classification do not overlap at all with the features used for subject identification. The first feature overlap occurs at top-50, and the third at top-70, meaning that 4% of the 70 most important ordinal pattern features are shared between the two models. The distribution of *D*, τ and *q* among the top-50 is shown in Figure 7. This implies that at the top of their respective ranking lists, the two classifiers rely on different features of the feature space. In other words, these results suggest that while both signatures coexist within the global feature space, the critical dimensions driving each task are separate, pointing toward a partial and layer-specific disentanglement between identity and biomarker information.

To contextualize the subject-identification performance, the achieved classification accuracy of 63.9% must be evaluated against the theoretical chance-level baseline. In a closed-set identification task encompassing N=31 subjects with balanced classes, the expected accuracy for a random classifier is approximately 3.23%. The observed performance represents an increase of nearly twenty times above chance level. Furthermore, this classification strength is supported by a robust Cohen’s kappa coefficient of κ=0.627, confirming that the individual identification is highly substantial and cannot be attributed to artifactual or random classification agreements.

A deeper inspection of the subject-identification performance reveals specific structural behaviors within the feature space. As illustrated in the normalized confusion matrix (Figure 5), the classification success is characterized by a remarkably uniform and solid diagonal across all 31 individuals. This high homogeneity demonstrates that the individual neurophysiological fingerprint is an intrinsic, stable property shared across the entire cohort, rather than an artifact driven by a small subset of highly distinct or outlier subjects. Furthermore, the distribution of cumulative relative feature importance (Figure 6) physically validates this task separation. At the very top of the ranking lists (N≤50), the classifiers rely on entirely mutually exclusive sets of features, meaning that the core predictive boundaries for identity and sleep-stage tracking are distinct. The feature overlap only increases significantly within the larger, lower-ranked feature subsets (N≥500), which primarily capture baseline broadband signal characteristics. Taken together, these figures empirically demonstrate that the feature space is functionally layered, allowing the machine learning architectures to untangle individual trait stability from transient macrostructural sleep biomarkers.

### 3.3. Ablation Analysis: Removing Subject-Informative Features

**Figure 8:**
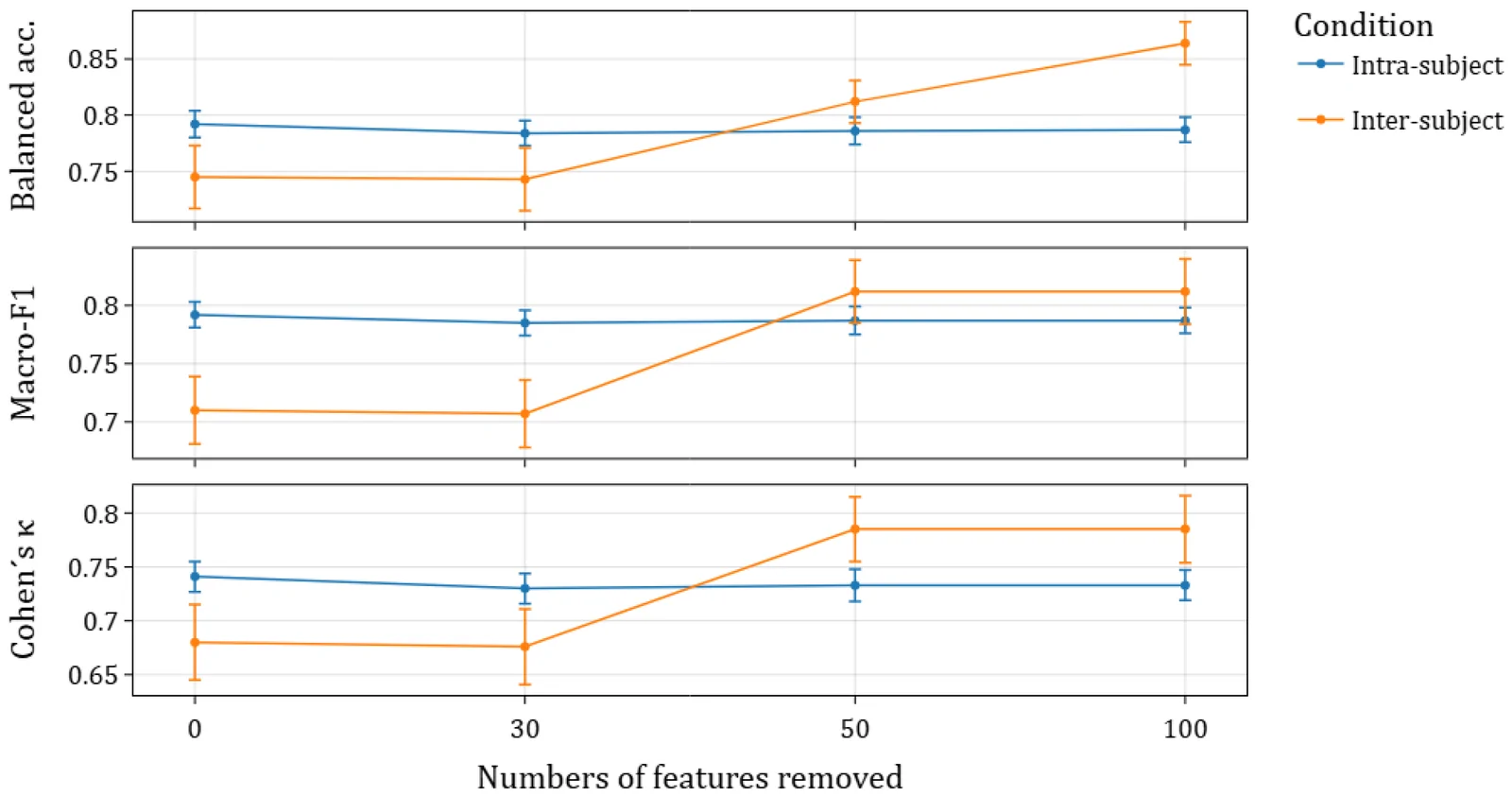
Mean sleep stage classification performance for the intra- and inter-subject conditions as a function of the number of top subject-informative features removed from the GWOP feature set. Error bars indicate the standard deviation across the 24 trials.

To further assess whether the sleep-stage classifier relies on subject-specific information, we performed an ablation analysis in which the most informative features for subject identification were systematically removed before training. First, the 3150 GWPO features were ranked according to their mean absolute SHAP values obtained from the subject-identification classifier. New sleep-stage classifiers were then trained after removing the top 30, 50, and 100 subject-informative features. Each ablated model was evaluated under both intra-subject and inter-subject validation schemes using the leave-group-out procedure described in Section 2.3.1. The corresponding performance metrics are reported in Table 11. To facilitate comparison with the baseline model trained using the complete GWPO feature set, Figure 8 summarizes the mean classification performance obtained after progressively removing the top subject-informative features.

## 4. Discussion

Generalized weighted ordinal-pattern (GWOP) features extracted from sleep EEG signals can support accurate sleep-stage classification while minimizing the detrimental effects of EEG fingerprinting and identity confounding. However, and although GWOP features contain sufficient information to discriminate individual subjects, they also preserve robust sleep-stage representations that generalize well to previously unseen individuals. Taken together, these findings suggest that fingerprinting and biomarker extraction should not necessarily be viewed as competing objectives, but rather as complementary information embedded within the same neurophysiological signal. A primary concern motivating this work was the possibility that sleep-stage classification performance could be artificially inflated by subject-specific information. Consistent with previous reports on EEG fingerprinting, the subject-identification analysis demonstrated that GWOP features retain substantial individual information, achieving an accuracy of 63.9% across 31 subjects, a value far above the expected chance level of 3.2%. This provides evidence that the GWOP feature space preserves highly discriminative, stable individual traits embedded within the sleep EEG dynamics, even when ignoring traditional macrostructural sleep-stage labeling. An identification accuracy of 63.9% across 31 subjects under a single EEG channel configuration is highly remarkable when compared with the current state of the art in neuroimaging and electrophysiology. In computational neuroscience, particularly in functional MRI (fMRI) connectome fingerprinting or high-density EEG resting-state spectral analyses involving small to moderate cohorts, similar single-session or single-channel identification rates are considered indicative of a highly potent, individualized neural signature. Given that sleep recordings are inherently non-stationary and dynamically shifting across radically different physiological macro-states, the fact that a single channel (Fpz-Cz) preserves such a pronounced individual signature underscores the capacity of ordinal-pattern representations to capture stable, idiosyncratic traits embedded within broader neural dynamics. The efficacy of the GWOP framework in person authentication stems from its ability to retain amplitude fluctuation dynamics through the entropic index *q*. While standard unweighted permutation entropy (q=0) treats low-intensity background noise and high-amplitude synchronized waves identically, the inclusion of generalized weights allows the model to capture personalized micro-structures. Specifically, positive *q* values accentuate robust, individualized macro-structures (such as characteristic delta wave bursts in N3), whereas negative *q* values isolate baseline noise or unique transient signatures during REM micro-arousals. The results imply that the exact distribution of these multi-scale amplitude variations forms a highly specific, idiosyncratic neural signature (an EEG fingerprint) that remains remarkably stable across subjects and sleep states.

However, if sleep-stage classification performance were primarily driven by such fingerprinting effects, a marked degradation would be expected when moving from intra-subject to inter-subject validation. Although the reduction was statistically significant (Wilcoxon signed-rank test, p<0.001), the observed differences ranged from 3.4 to 8.4 percentage points across the evaluated metrics. Mean classification accuracy decreased by only 3.4 percentage points, while balanced accuracy decreased by 4.8 percentage points. This moderate performance drop observed from intra to inter-subject condition (ΔF1=0.084) may be attributable to natural physiological between-subject variability rather than to fingerprinting. It is critical to address the methodological choice regarding class balancing across the validation schemes. While the intra-subject evaluation utilized an artificially balanced dataset to force the XGBoost classifier to optimize for subtle morphological boundaries (such as N1 versus REM) rather than majority class prevalence, the inter-subject testing intentionally retained the natural, unbalanced physiological distribution of sleep stages. Consequently, part of the reported performance gap (ΔAccuracy=3.4%, ΔMacroF1=0.084) is likely driven by this shift in the operational label distribution rather than purely reflecting a detrimental cross-subject fingerprinting effect. Testing on the raw, skewed biological base-rates provides a more rigorous and realistic assessment of the model’s clinical generalizability, demonstrating that the GWOP representations remain highly effective even when facing the intrinsic class imbalances of real-world overnight polysomnography. The GWOP features that drive classification, particularly those with D=5 and τ=3, appear to encode genuinely generalizable sleep stage information. Moreover, confusion matrices remained qualitatively similar across validation schemes, indicating that the classifier learned sleep-stage representations that largely generalized to unseen subjects.

The most important features under SHAP analysis for sleep-stage classification were virtually identical under intra-subject and inter-subject evaluation. The overlap between the top-ranked features exceeded 98% across a broad range of feature-set sizes, and the complete SHAP importance vectors exhibited an almost perfect correlation. Within the top-30 most important features, complete agreement was observed between conditions. Divergence only emerged at larger *N*, reaching 2.4% at top-500 (12 out of 500 features). This pattern, combined with the Spearman correlation of ρ=0.998 across the full feature vector, provides strong evidence that the classifier does not exploit subject-specific signals to achieve its intra-subject performance. The moderate between-trial stability of SHAP rankings (ρ≈0.43) further reflects the expected variability arising from differences in subject group composition across trials, and is not indicative of a systematic fingerprinting effect. Also, the mean correlation was virtually identical between the intra- and inter-subject conditions (difference of 0.005, well within one standard deviation of 0.044). This indicates that the trial-to-trial variability in feature rankings is not systematically affected by whether the model is evaluated on seen or unseen subjects. If fingerprinting were present, intra-subject rankings would be expected to be substantially more stable than inter-subject rankings, as the model would consistently exploit subject-specific signals when predicting on known subjects. The absence of such a difference provides further evidence against the presence of fingerprinting in the GWOP feature set. This implies that classifiers rely on the same underlying physiological signatures regardless of whether subjects were previously observed during training.

Perhaps the most important result emerges from the comparison between sleep-stage classification and subject-identification models. Despite both analyses using the same GWOP feature space, the highest-ranked features for the two tasks showed almost no overlap. In fact, among the 100 most important features, only a small number contributed substantially to both models. This dissociation suggests that GWOP representations encode multiple forms of information simultaneously, with some features preferentially capturing sleep-stage dynamics and others reflecting stable individual characteristics. From a machine-learning perspective, this observation implies a partial orthogonality between biomarker-related and identity-related information. Such a property is highly desirable because it enables the extraction of clinically meaningful biomarkers without necessarily eliminating the individualized structure inherent to EEG signals. From a neurophysiological standpoint, these findings are consistent with emerging views that sleep EEG activity contains multiple layers of organization operating at different temporal and biological scales. Sleep stages reflect transient global brain states characterized by distinct oscillatory and dynamical regimes, whereas EEG fingerprints likely emerge from stable anatomical, physiological, and network-level properties of individual brains. Ordinal-pattern representations appear capable of capturing both dimensions simultaneously. This may explain why the same feature framework can support accurate sleep-stage classification while also enabling subject recognition.

From a methodological perspective, the existence of this prominent subject-specific signature does not degrade or compromise sleep-stage classification performance in the intra-subject condition due to the partial orthogonality of the feature space. As revealed by the SHAP feature-importance analysis, the specific GWOP that drive individual recognition do not overlap with those governing sleep-state transitions at the top of their respective rankings. Although feature overlap increases as larger sets of features are considered—reaching 13% at the top-100 and 39% at the top-500—the absolute zero overlap observed among the most critical features (N<30) implies a functional orthogonality at the core of the models’ decision-making boundaries. For the most critical features (N<30), the overlap is strictly zero, implying that the machine learning models utilize entirely different microstructural properties of signals for each objective. While identity classification primarily leverages multi-scale amplitude variances (captured by specific combinations of non-zero entropic indices *q*), sleep-stage scoring depends on the raw temporal ordering and dominant state-dependent macrostructural patterns. This mathematical separation prevents identity-related data leakage from inflating or confounding the physiological features required for generalizable biomarker extraction. However, the absence of structural overlap does not preclude subject-informative features from affecting classification performance. The ablation analysis shows that removing the top subject-informative features substantially improves inter-subject performance, with Macro F1 increasing from 0.708±0.029 to 0.812±0.028, while intra-subject performance remains stable (0.792±0.011 vs. 0.787±0.011). This occurs because subject-informative features do not compete with sleep-stage features in the importance ranking, but they do generate signal features that the model cannot use when facing a new subject. During training, the model learns identity-related patterns from known subjects. These patterns do not transfer to unseen individuals. As a result, they act as confounding information that impairs generalization. In the intra-subject condition, by contrast, the model already knows the subject and can compensate for this signal, which is why its removal produces no change in performance.

## 5. Conclusions

We presented a comprehensive study on the use of ordinal-pattern-based analysis in sleep EEG characterization. The presented results demonstrate that GWOP features simultaneously encode individualized and sleep-stage-specific information. However, rather than complete disentanglement, our findings support a model of partial separation within the latent space, where individual identity features are highly robust but operate independently from the primary biomarker dimensions at the top of the feature ranking. Rather than constituting an unavoidable source of confounding, EEG fingerprinting may coexist with clinically relevant biomarkers in a manner that permits reliable generalization across subjects. The capacity to characterize an individual’s baseline neural signature enhances the sensitivity of biomarker detection by establishing a personalized reference frame. This individualized benchmark allows pathological deviations—whether functional or structural—to be measured relative to a subject’s own normative state rather than against heterogeneous population averages. This distinguishes stable trait-related information from disease-related effects, while granting that predictive models generalize to previously unseen individuals. These results show that GWOP features can successfully preserve generalizable physiological biomarkers without requiring the suppression of the individual neurophysiological footprint. This can lead to further research focused on other fruitful goals in clinical aspects of disease tracking. This concept is closely aligned with emerging precision medicine approaches that emphasize within-subject trajectories rather than solely population-level comparisons. Our findings support the use of ordinal-pattern-based complexity measures as a promising framework for sleep EEG analysis. Although our current dataset does not include clinical or longitudinal outcomes to empirically demonstrate personalized diagnostic tracking, we hypothesize that future biomarker discovery efforts may benefit from explicitly modeling, rather than suppressing, the individualized nature of brain dynamics. From a speculative standpoint, this perspective could guide the development of future frameworks capable of jointly representing identity and pathology, allowing researchers to quantify their respective contributions to EEG variability. Under these conditions, the ability to characterize an individual’s baseline neural signature can enhance the sensitivity of biomarker detection by providing a personalized reference against which pathological deviations can be measured.

Several limitations should nevertheless be acknowledged. The present study is framed primarily as a proof-of-concept investigation, given that the analysis was restricted to a single EEG derivation and a relatively homogeneous cohort of healthy young adults from a single database. Additional studies involving larger populations, more diverse age brackets, multiple recording sites, and clinically diagnosed sleep disorders will be necessary to determine whether the observed separation between fingerprinting and biomarker information remains stable under more heterogeneous conditions and to fully validate its generalizability in clinical practice. Furthermore, although SHAP analysis provides valuable insights into feature importance, it does not directly characterize the geometric structure of the latent feature space. Future work could employ representation-learning approaches, manifold analysis, or information-theoretic frameworks to quantify the degree of independence between subject-specific and sleep-related information.

## Figures and Tables

**Table 1:** Number of GWOP features extracted for each parameter configuration.

D	OPs	τ	*q* Values	Features
3	6	1, 2, 3	−1,0,1,2,3,4,5	126
4	24	1, 2, 3	−1,0,1,2,3,4,5	504
5	120	1, 2, 3	−1,0,1,2,3,4,5	2520
			Total	3150

**Table 2:** Mean classification performance across 24 trials under intra-subject and inter-subject test conditions, together with the bootstrap 95% confidence intervals for Δ and the *p*-values from the Wilcoxon signed-rank test.

Metric	Intra-Subject	Inter-Subject	Δ	95% IC of Δ	Wilcoxon *p*
Acc.	0.792±0.011	0.758±0.031	+0.034	[0.020, 0.050]	<0.001
Balanced acc.	0.792±0.011	0.744±0.027	+0.048	[0.035, 0.061]	<0.001
Macro F1	0.792±0.011	0.708±0.029	+0.084	[0.070, 0.099]	<0.001
Cohen’s κ	0.740±0.014	0.679±0.036	+0.061	[0.044, 0.080]	<0.001

**Table 3:** Mean precision, recall and F1-score per sleep stage across 24 trials under intra-subject and inter-subject test conditions. Values are reported as mean ± standard deviation.

Stage	Condition	Precision	Recall	F1
W	Intra	0.895±0.017	0.865±0.020	0.879±0.013
	Inter	0.913±0.052	0.812±0.091	0.855±0.050
N1	Intra	0.643±0.026	0.651±0.037	0.646±0.027
	Inter	0.292±0.059	0.619±0.107	0.390±0.050
N2	Intra	0.788±0.025	0.785±0.029	0.786±0.019
	Inter	0.880±0.051	0.759±0.072	0.812±0.044
N3	Intra	0.897±0.017	0.913±0.017	0.905±0.013
	Inter	0.746±0.109	0.851±0.074	0.787±0.058
R	Intra	0.742±0.029	0.745±0.034	0.743±0.026
	Inter	0.748±0.107	0.678±0.120	0.698±0.072

**Table 4:** Distribution of the optimal XGBoost hyperparameters across the 24 trials for sleep-stage classification models. Values are reported as mean ± standard deviation.

Parameter	Mean Value ± Standard Deviation
Learning rate	0.223±0.040
Maximum depth	5.96±1.12
Number of trees	120.46±10.29
Minimum child weight	2.42±0.78
Column subsampling	0.204±0.079
Row subsampling	0.886±0.059
Gamma	0.077±0.093
L1 regularization (α)	0.116±0.065
L2 regularization (λ)	2.007±0.179

**Table 5:** Overlap between top-*N* intra-subject and inter-subject SHAP feature sets. Fingerprint features are those appearing in the intra ranking but not in the inter ranking.

Top-*N*	Overlap	Diff
10	10 (100%)	0
20	20 (100%)	0
30	30 (100%)	0
50	49 (98%)	1
100	99 (99%)	1
200	197 (98%)	3
500	488 (98%)	12
1000	979 (98%)	21
1500	1468 (98%)	32
2000	1971 (99%)	29
2500	2476 (99%)	24
3000	2985 (100%)	15

**Table 6:** Between-trial stability of SHAP feature rankings, measured as pairwise Spearman rank correlation across the 24 trials.

Condition	Mean ρ	SD
Intra-subject	0.437	0.044
Inter-subject	0.432	0.044

**Table 7:** Top-10 GWOP features ranked by global mean absolute SHAP value. Values are identical under intra- and inter-subject conditions.

Rank	Feature				SHAP_intra_	SHAP_inter_
	Pattern	D	τ	q		
1	[42103]	5	3	+1	0.0698	0.0698
2	[10324]	5	2	+1	0.0467	0.0467
3	[10234]	5	1	0	0.0425	0.0425
4	[201]	3	2	0	0.0423	0.0423
5	[20314]	5	2	−1	0.0362	0.0362
6	[30124]	5	3	+1	0.0357	0.0357
7	[10234]	5	3	−1	0.0356	0.0356
8	[42103]	5	3	+2	0.0342	0.0342
9	[43201]	5	1	+1	0.0317	0.0317
10	[43201]	5	1	0	0.0311	0.0311

**Table 8:** Performance of the subject-identification classifier using GWOP and spectral features representations.

Feature Representation	Target	Accuracy	Macro-F1	Cohen’s κ
GWOP	Subject	0.639	0.638	0.627
Spectral features	Subject	0.432	0.428	0.413

**Table 9:** Optimal XGBoost hyperparameters obtained through random search for subject identification.

Parameter	Value
Learning rate	0.201
Maximum depth	4
Number of trees	128
Minimum child weight	3
Column subsampling	0.251
Row subsampling	0.924
Gamma	0.162
L1 regularization (α)	0.208
L2 regularization (λ)	2.321

**Table 10:** Overlap between top-*N* SHAP feature sets for sleep-stage classification and subject identification. Features appearing in the sleep-stage ranking but not in the subject-identification ranking indicate that the classifier relies on sleep-specific information rather than subject identity.

Top-*N*	Overlap	Only in Subject Classification
30	0 (0%)	30 (100%)
40	0 (0%)	40 (100%)
50	1 (2%)	49 (98%)
60	1 (2%)	59 (98%)
70	3 (4%)	67 (96%)
80	6 (8%)	74 (92%)
90	8 (9%)	82 (91%)
100	13 (13%)	87 (87%)
200	39 (20%)	161 (80%)
300	91 (30%)	209 (70%)
400	138 (35%)	262 (65%)
500	197 (39%)	303 (61%)
1000	553 (55%)	447 (45%)
1500	987 (66%)	513 (34%)
2000	1492 (75%)	508 (25%)
2500	2114 (85%)	386 (15%)
3150	3150 (100%)	0 (0%)

**Table 11:** Mean classification performance under intra-subject and inter-subject validation conditions as a function of the number of top subject-informative features removed from the GWOP feature set. Values are reported as mean ± standard deviation across the 24 trials.

Removed	Condition	Bal. Acc.	Macro F1	Cohen’s κ
30	Intra	0.784±0.011	0.785±0.011	0.730±0.014
	Inter	0.743±0.028	0.707±0.029	0.676±0.035
50	Intra	0.786±0.012	0.787±0.012	0.733±0.015
	Inter	0.812±0.019	0.812±0.027	0.785±0.030
100	Intra	0.787±0.011	0.787±0.011	0.733±0.014
	Inter	0.864±0.019	0.812±0.028	0.785±0.031

## Data Availability

The data analyzed in this study are publicly available from the Sleep-EDF Database Expanded through PhysioNet[https://physionet.org/content/sleep-edfx/1.0.0/], accessed on 23 July 2026. The models generated during the current study are available from the corresponding author upon reasonable request.
